# Modification of the Surface Topography and Composition of Ultrafine and Coarse Grained Titanium by Chemical Etching

**DOI:** 10.3390/nano7010015

**Published:** 2017-01-13

**Authors:** Denis V. Nazarov, Elena G. Zemtsova, Alexandr Yu. Solokhin, Ruslan Z. Valiev, Vladimir M. Smirnov

**Affiliations:** Saint Petersburg State University, 7/9 Universitetskaya nab., St. Petersburg 199034, Russia ; ezimtsova@yandex.ru (E.G.Z.); solohin150194@mail.ru (A.Y.S.); rzvaliev@gmail.com (R.Z.V.); vms11@yandex.ru (V.M.S.)

**Keywords:** chemical etching, UFG titanium, surface, roughness, titanium implants

## Abstract

In this study, we present the detailed investigation of the influence of the etching medium (acidic or basic Piranha solutions) and the etching time on the morphology and surface relief of ultrafine grained (UFG) and coarse grained (CG) titanium. The surface relief and morphology have been studied by means of scanning electron microscopy (SEM), atomic force microscopy (AFM), and the spectral ellipsometry. The composition of the samples has been determined by X-ray fluorescence analysis (XRF) and X-ray Photoelectron Spectroscopy (XPS). Significant difference in the etching behavior of UFG and CG titanium has been found. UFG titanium exhibits higher etching activity independently of the etching medium. Formed structures possess higher homogeneity. The variation of the etching medium and time leads to micro-, nano-, or hierarchical micro/nanostructures on the surface. Significant difference has been found between surface composition for UFG titanium etched in basic and acidic Piranha solution. Based on the experimental data, the possible reasons and mechanisms are considered for the formation of nano- and microstructures. The prospects of etched UFG titanium as the material for implants are discussed.

## 1. Introduction

Titanium and its alloys have a unique combination of mechanical properties (hardness, strength, low density, and relatively low Young modulus) and excellent biocompatibility [1,2]. This allows it to be widely used as the most suitable material for orthopedic and dental implants [1,2,3]. The alloys provide enhanced mechanical properties of the material, however, they are potentially dangerous due to possible release of allergens and toxic elements [4]. The most suitable alternative is to use pure titanium in the ultrafine grained (UFG) forms, i.e., with the grain size between tens and hundreds nanometers [5,6,7]. UFG metals (particularly, titanium) are expected to have more implant-suitable mechanical properties (high fatigue strength, tensile strength, and low Young modulus value) as compared to coarse grained (CG) analogs [5,7,8]. Therefore, UFG-based implants provide better reliability and durability. In addition to the mechanical properties, UFG structure can promote adhesion, spreading, proliferation, differentiation of bone tissue cells, and also accelerated tissue mineralization [9], which eventually promotes the implant’s engraftment. In turn, acceleration of the implant’s engraftment is the most important and the most complicated task in the development of the new generation of implants [1,2].

Nevertheless, according to the data reviewed in [9], the enhancement of the biomedical properties of UFG materials compared to CG analogs is not valuable. This is not sufficient for the meaningful acceleration of the implant’s engraftment, reliability, and improvement of biocompatibility. In this regard, additional surface modification is required. Necessary surface relief is developed by means of electrochemical anodization [10,11], sand blasting [12,13], and chemical etching [12,13,14,15]. Alternatively, bioactive coatings are deposited by means of chemical vapor deposition (CVD), physical vapor deposition (PVD), sol-gel, ionic implantation [13].

Among the above-mentioned methods, chemical etching is currently the most interesting one due to its simplicity combined with wide possibilities to variate both relief and composition of the surface [14,15]. At the moment, numerous experimental studies are known on the etching of CG titanium and its alloys in various etching media [13,14,15,16]. Oppositely, despite the wide prospects of UFG titanium and great scientific interest in it, the UFG titanium etching is still not studied enough. There are some works dedicated to UFG titanium corrosion [17,18,19,20] that suggest significant differences between rate, character, and mechanism of corrosion of UFG and CG titanium. One can expect the differences also in the case of etching. So, we demonstrated earlier using mass loss analysis [21] that the rate of etching of UFG titanium in Piranha solutions is significantly higher than for CG titanium. Similar results were described in the work [22], where solutions of HF and HF/HNO_3_ were used as etching media.

The current study is devoted to the more detailed investigation of the etching features of UFG and CG titanium in acidic (H_2_SO_4_/H_2_O_2_) and basic (NH_4_OH/H_2_O_2_) Piranha solutions. Namely, changes of relief and surface composition during etching are described; optimal conditions of UFG titanium etching are specified in order to make the material most suitable for new generation implants.

## 2. Results

### 2.1. Morphology of the Surface

#### 2.1.1. H_2_SO_4_/H_2_O_2_ Etching

The surface morphology of etched UFG and CG titanium has been studied by scanning electron microscopy (SEM). Micrographs with magnification from 300 to 600,000 have been used for morphology analysis both on the nano- (600,000–100,000×) and microscale (10,000–300×).

**Figure 1:**
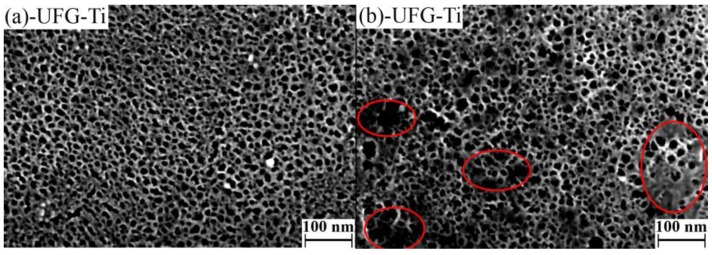
Characteristic scanning electron microscopy (SEM) images of ultrafine grained-ultrafine grained (UFG) (**a**) and coarse grained-coarse grained (CG) (**b**) titanium etched in H_2_SO_4_/H_2_O_2_ solutions during 2 h (magnification—400,000×). Red lines mark inhomogeneous areas.

**Figure 2:**
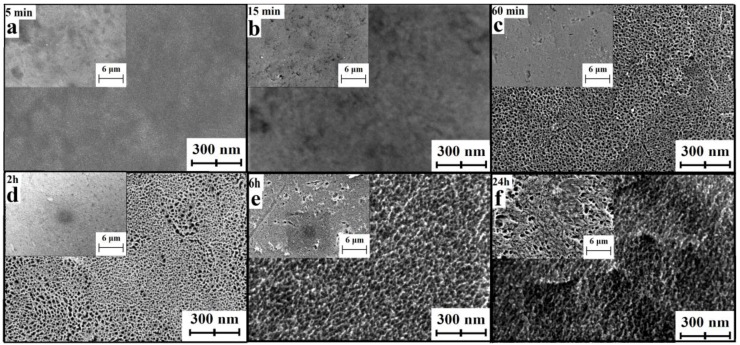
Characteristic SEM images of UFG titanium etched in H_2_SO_4_/H_2_O_2_ solutions during 5 (**a**), 15 min (**b**); and 1 (**c**), 2 (**d**), 6 (**e**) and 24 h (**f**) (magnification 200,000×—main picture, 10,000×—insets).

UFG titanium etching in H_2_SO_4_/H_2_O_2_ during 5 and 15 min does not influence on morphology, but our previous results [21] suggest significant mass loss of the sample. Therefore, we can conclude that within this time interval, layer by layer etching with diffusion control takes place. More prolonged treatment in the etching medium leads to “sponge-like” structure on the nanoscale (Figure 1 and Figure 2). This change can be explained only by the difference of the etching rate of various spots of the surface. In this case, the etching takes place with kinetic control. When the time of etching increases to 2 h, the “sponge-like” structure becomes more homogeneous; after more prolonged treatment the structure is densified. It is worth noting that the image’s contrast becomes worse. It can be caused by material oxidation during long-term etching.

**Figure 3:**
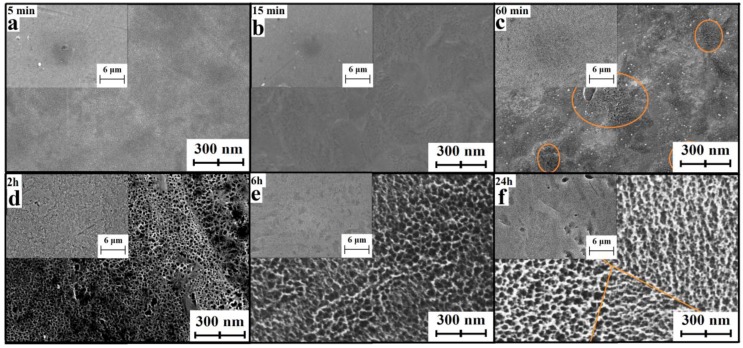
Characteristic SEM images of СG titanium etched in H_2_SO_4_/H_2_O_2_ solutions during 5 (**a**), 15 min (**b**); and 1 (**c**), 2 (**d**), 6 (**e**) and 24 h (**f**) (magnification 200,000×—main picture, 10,000×—insets). Orange lines mark various etching areas.

In the case of CG titanium, the surface morphology changes become visible already after 15 min etching (Figure 3). “Sponge-like” structure is locally appeared after 1 h (Figure 3—in orange circles), but fully covers the sample surface only after 2 h treatment. After 24 h etching, sponge morphology varies depending on the location on the surface (Figure 3—differing regions are separated by orange lines). Supposedly, this change relates to the structure of CG Ti (i.e., the presence of the micron-sized grains and their boundaries).

SEM micrographs of smaller magnification (Figure 2 and Figure 3 insets) do not demonstrate significant morphology changes in the microscale while etching in the acidic Piranha solution except pits with diameters of 1–3 µm. Such pits are typical for the samples after 6 and 24 h treatment UFG titanium and after 24 h treatment CG titanium. Moreover, the amount of pits is greater on the UFG titanium surface. Analysis of the large series of SEM microphotographs with different magnifications revealed more significant inhomogeneity of nano- and microstructures formed in etched CG titanium compared to UFG titanium. The examples of typical homogeneous and inhomogeneous surfaces of UFG and CG Ti are shown in Figure 1.

#### 2.1.2. NH_4_OH/H_2_O_2_ Etching

**Figure 4:**
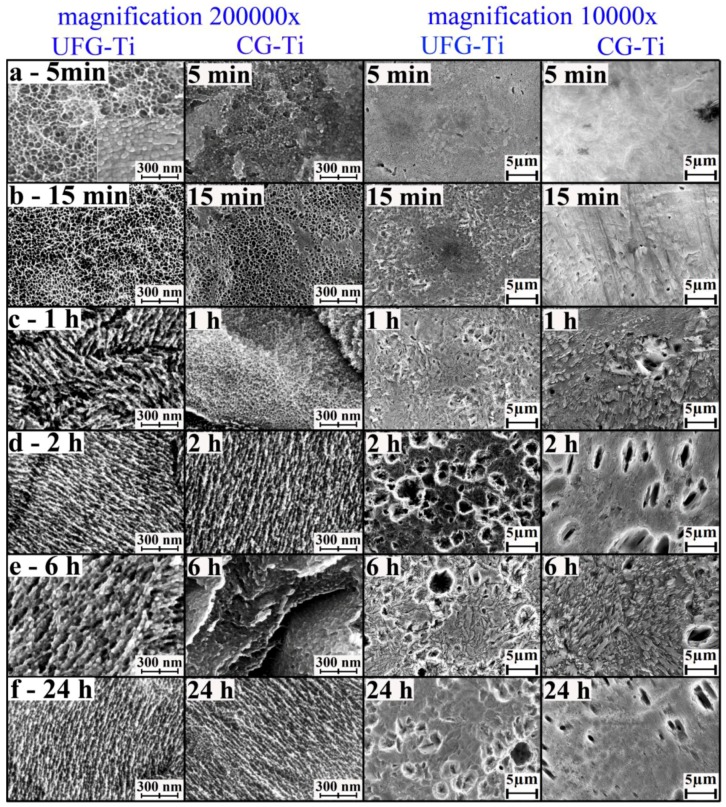
Characteristic SEM images of UFG and СG titanium etched in H_2_SO_4_/H_2_O_2_ solutions during 5 (**a**), 15 min (**b**); and 1 (**c**), 2 (**d**), 6 (**e**) and 24 h (**f**) (magnifications 200,000× and 10,000×).

Etching of UFG titanium in NH_4_OH/H_2_O_2_ solution leads to the significant mass loss [21] and surface morphology variation in the nanoscale even on the early etching stages (Figure 4). The sample after a 5 min etching has the developed relief, however, it is not homogenous on the whole sample surface. Some surface spots are “mesh-like” whereas other spots consist of densely located particles with diameters of tens of nanometers. 15 min etching gives the network structure. More prolonged etching leads to the formation of shortened “ridges”—such structure can be called “coral-like” [23].

Etching of CG titanium is similar to that of UFG titanium. However, network elements appeared only after 15 min treatment and “coral-like” structure is found after 2 h etching.

Less magnified SEM images also demonstrate significant morphology change in microscale on the very first etching stages. The most valuable changes for both titanium types are found after 2 h treatment: micron-sized pits become evident. However, for CG titanium, the pits are elongated and their number is much less. Besides, “lamellar” formations can be found on CG titanium surface, especially for the sample etched for 6 h. It is worth noting that the UFG sample after 6 h of etching is also slightly different by morphology as “ridges” become larger in size.

### 2.2. Relief of the Surface

#### 2.2.1. H_2_SO_4_/H_2_O_2_ Etching

**Figure 5:**
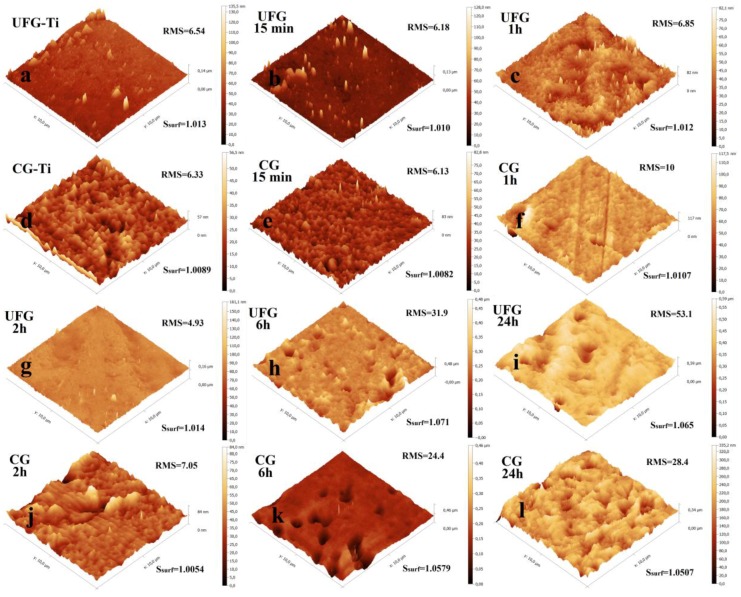
Atomic force microscopy (AFM) surface topographies of the UFG and CG titanium etched in H_2_SO_4_/H_2_O_2_. Nonetched UFG-Ti (**a**), UFG-Ti etched 15 min (**b**), 1 h (**c**), nonetched CG-Ti (**d**), CG-Ti etched 15 min (**e**), 1 h (**f**), UFG-Ti etched 2 h (**g**), 6 h (**h**), 24 h (**i**), CG-Ti etched 2 h (**j**), 6 h (**k**), 24 h (**l**).

The surface relief of the etched UFG and CG titanium has been studied by AFM. The surface topographies were measured with the scales of 50 × 50, 30 × 30, 10 × 10 (Figure 5) and 1 × 1 µm. The task of quantitative characterization of relief is rather complicated [24,25], so we calculated the parameters of the arithmetic mean (*R*_a_) and root mean square (RMS) roughness, maximal height amplitudes (*R*_max_), and specific surface area (*S*_surf_). These parameters provide the most full quantitative description of the surface relief, because the values of *R*_a_ and RMS work very well for robust characterization of overall roughness of etched isotropic surfaces, *R*_max_ is very sensitive to noise, defects, and spikes; and *S*_surf_ shows the degree of development of the surface [24].

**Figure 6:**
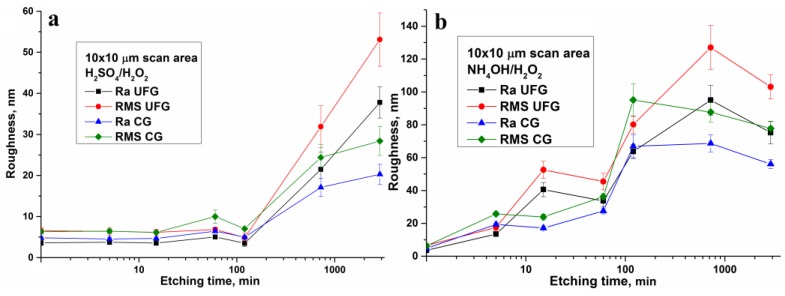
Roughness values as a function of the etching time for solutions H_2_SO_4_/H_2_O_2_—(**a**) and NH_4_OH/H_2_O_2_—(**b**).

**Figure 7:**
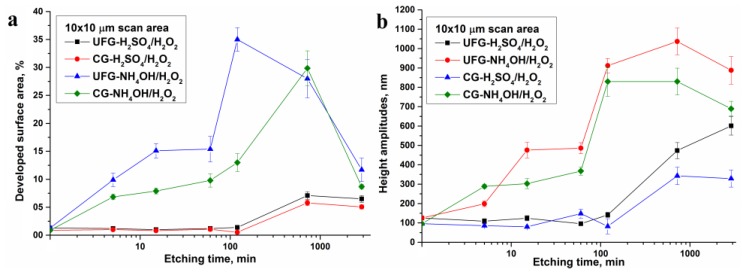
Specific surface area (**a**) and height amplitudes (**b**) as a functions of the etching time.

Calculated relief parameters for the scans 1 × 1 and 10 × 10 µm are given in the Tables S1–S4 and also represented graphically in Figure 6 and Figure 7. The relief parameters (roughness—Figure 6a, specific surface area—Figure 7a, height amplitudes—Figure 7b) slowly increase both in nano (1 × 1 µm scans) and in micro scale (10 × 10 µm scans) while etching time increased from 5 min to 2 h. The changes are not monotonic. In the micro scale, a jump in roughness is observed for etching times between 2 and 6 h. In this interval, the values *R*_a_, RMS, *R*_max_, and *S*_surf_ for 10 × 10 µm scans are changed from 3.50, 4.93, 142, and 1.014 to 21.5, 31.9, 474, and 1.072 for UFG titanium. Analogous values for CG titanium increase from 5, 7.1, 83, and 1.005 to 17.1, 24.4, 343, and 1.058. A less pronounced parameter jump is observed also for 1 × 1 µm scans. The analysis of 3D surface topographies (Figure 5) together with SEM images (Figure 2 and Figure 3) suggests that this jump is caused both by micron-sized pits and by nanoscale changes in the surface morphology.

Values of roughness, height amplitude, and surface area of UFG titanium are meaningfully higher than the analogous values for CG titanium. This fact confirms higher activity of nanostructured material while etching.

#### 2.2.2. NH_4_OH/H_2_O_2_ Etching

**Figure 8:**
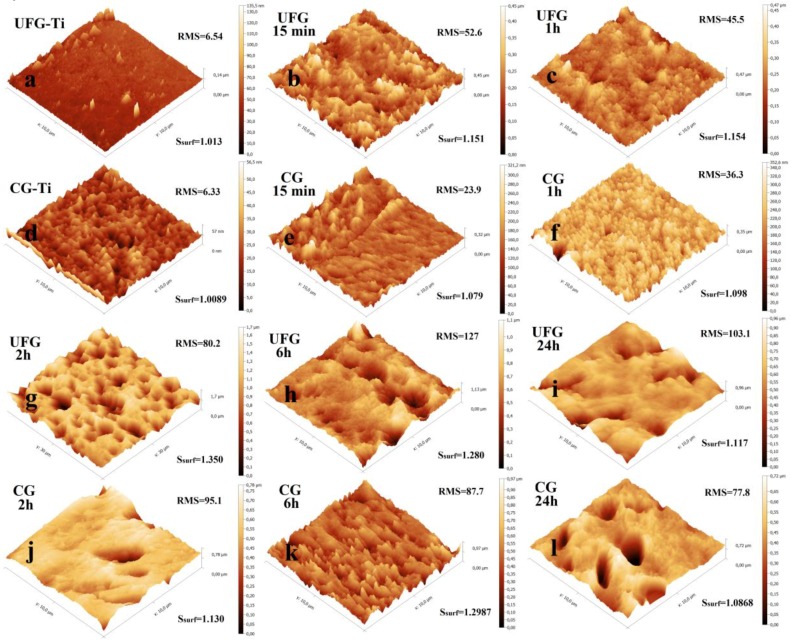
AFM surface topographies of the UFG and CG titanium etched in NH_4_OH/H_2_O_2_. Nonetched UFG-Ti (**a**), UFG-Ti etched 15 min (**b**), 1 h (**c**), nonetched CG-Ti (**d**), CG-Ti etched 15 min (**e**), 1 h (**f**), UFG-Ti etched 2 h (**g**), 6 h (**h**), 24 h (**i**), CG-Ti etched 2 h (**j**), 6 h (**k**), 24 h (**l**).

Etching in NH_4_OH/H_2_O_2_ solution leads to significant increase of topography parameters even on the earliest etching stages. However, these values are changed non-monotonically with increasing etching time. Between 5 and 15 min, a sharp increase of *R*_a_, RMS, and *R*_max_ values is observed for UFG titanium only (Figure 6b and Figure 7b). This is due to the qualitative modification of the surface morphology. The values *R*_a_, RMS, and *R*_max_ are significantly increased also in the etching time interval between 1 and 2 h both for UFG and CG titanium. In the same time interval, a sharp increase of *S*_surf_ from 15.4% to 35% is observed for UFG titanium (Figure 7a). AFM 3D surface topographies for UFG and CG titanium (Figure 8) demonstrate the formation of pits at etching times of 2 h or more. More prolonged etching times (6 and 24 h) lead to a decrease of specific surface area of UFG titanium. Maximal *S*_surf_ value for CG titanium (29.9%) is reached only after 6 h etching. It is worth noting that according to AFM topographies (Figure 8) and SEM images (Figure 4), this sample differs from others by lamellar structure.

It is also important that ammonia Piranha solution etching for UFG titanium is quicker than for CG titanium (analogously to sulfuric acid Piranha solution) [21], and relief parameters are higher. For NH_4_OH/H_2_O_2_, the difference is sufficiently higher than for H_2_SO_4_/H_2_O_2_.

### 2.3. Composition of the Surface

The study of the composition of the etched and non-etched CG and UFG titanium by XRF have demonstrated that impurity content of the material was Fe—0.22–0.25, O—0.10–0.24, Cu—0.09–0.15 (wt %). The study of the samples surface composition by X-ray Photoelectron Spectroscopy (XPS) showed the presence of Ti, O, and C. The carbon contamination is seemingly caused by adventitious atmospheric hydrocarbon on the surface of the sample. No other contaminants of the samples’ surfaces were found.

**Figure 9:**
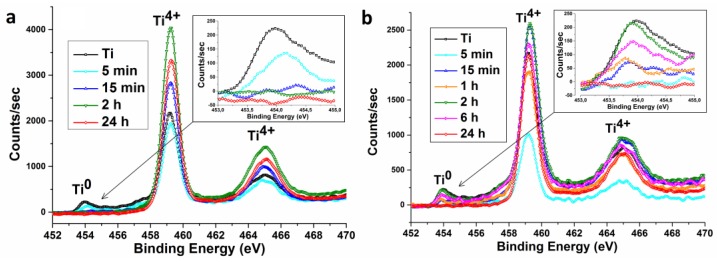
XPS high resolution Ti 2p spectra of nanotitanium etched in (**a**) acidic Piranha and (**b**) basic Piranha.

According to high resolution XPS spectra of Ti 2p (Figure 9), the Ti 2p_3/2_ and Ti 2p_1/2_ peaks are located at 459.2 eV and 464.9 eV for all samples and can be attributed to Ti^4+^ [26]. No Ti^3+^ or Ti^2+^ shoulders at lower binding energy are detected, suggesting that all samples have a stoichiometric TiO_2_ surface. Metallic Ti peak (453.9 eV) is quite evident in the spectrum of initial titanium. This peak is still found in the sample etched for 5 min in H_2_SO_4_/H_2_O_2_. However, the peak disappears when the time increases to 15 min and above (Figure 9a, inset). Since XPS allows to study only surface layer (several nm) of the material, peak Ti is disappeared, probably due to the oxidation. Titanium oxidation while acidic Piranha etching is confirmed by the ellipsometry data. Initial TiO_2_ layer thickness is estimated at 6 nm and this thickness grows to 45–50 nm when the etching time increases from 0 to 24 h.

The result is different while using the NH_4_OH/H_2_O_2_ etching medium. On the early etching stages (5 min) Ti peak disappears, but later this peak starts to grow, its intensity reaches maximum at 2 h etching. According to AFM and SEM data, this time is characterized by the formation of micron-size pits. Unfortunately, it is impossible to confirm variations of oxide layer thickness by means of ellipsometry. This is due to the significant roughness and light absorption by the samples’ surfaces. The plausible ellipsometric results for the TiO_2_ layer thickness have been obtained only for the samples etched for 5 and 15 min. The layer thicknesses were estimated as 15–25 nm.

**Figure 10:**
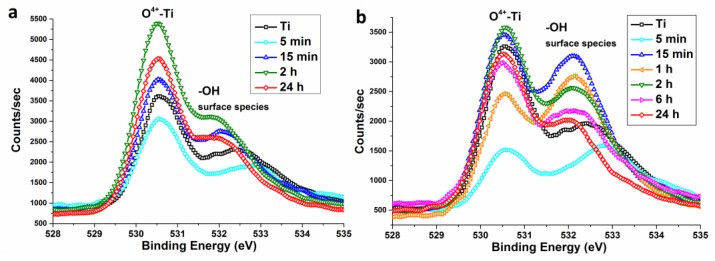
XPS high resolution O 1s spectra of nanotitanium etched in (**a**) acidic Piranha and (**b**) basic Piranha.

Both initial and Piranha treated Ti samples show an intensive O 1s peak from Ti–O bonds at 530.5 eV (Figure 10) and a second peak at higher energy which can be attributed to the contributions of –OH and H_2_O surface species [26]. Its intensity is not strongly changed after chemical treatment in H_2_SO_4_/H_2_O_2_. In turn, the samples of NH_4_OH/H_2_O_2_ series are characterized by meaningful variation of relative intensities of the peaks when etching time is increased. However, no distinct correlations between intensities and etching times were found. Relative intensities suggest that the samples etched in ammonia Piranha solution contain more functional groups than ones etched in acidic Piranha solutions. However, this difference can also be due to the variation of the specific surface area values and morphologies.

## 3. Discussion

### 3.1. Etching of UFG and CG Titanium

The processes that accompany the chemical etching are rather complicated. They involve material removal, oxidation, and surface passivation. These processes depend on etching medium and etching conditions (duration, concentration, and temperature). Depending on the conditions, etching obeys either diffusion or kinetic control. In the case of diffusion control, the processes are controlled by the rates of reagent supply and reaction product removal from the surface area. In this case, the etching is performed layer by layer; surface roughness is smoothed. The rate limiting stage for kinetic control reactions is the reaction of the local areas on the surface. In the case of surface inhomogeneity and meaningful difference of reaction ability of local areas, the significant change in relief is possible.

Data of this combined with data on the mass loss at the etching [21] suggest that etching by Piranha solutions can follow both abovementioned mechanisms. For H_2_SO_4_/H_2_O_2_ solution, within minutes or tens of minutes, diffusion control is realized. This leads to slow layer-by-layer etching and smoothing of surface defects. With more prolonged treatment (several h or more) etching can perform with kinetic control that leads to the formation of micron-sized pits. In ammonia Piranha solution, etching is performed with kinetic control in the first minutes. This leads to rapid modification of the surface relief.

In a general case, switching of the etching mechanism can be due to the variation of conditions (e.g., concentration, temperature, and mixing). In our work, the temperature remained unchanged, mixing has not been applied. Reagents’ concentrations are really decreased, but for this variation, switching from kinetic to diffusion control is expected. However, we observe either no changes (NH_4_OH/H_2_O_2_), or reverse effect (H_2_SO_4_/H_2_O_2_). Therefore, the changes in the etching mechanism are caused by the features and composition of the surface, but do not depend on changes in the process conditions. It is important to note that sample surfaces were mechanically treated (polished) before etching. Therefore, surface structure was modified. We assume that the surface structure of polished titanium is homogenous and isotropic. Such surface is etched uniformly and layer-by-layer. The difference in the etching rate for the different spots of the surface becomes visible only after removal of the surface layer and structured material layers appear on the surface. Thus, in the case of etching in sulfuric acid Piranha, removal of the surface layer is slow and we can observe the transition from diffusion to the kinetic control. In the case of etching in the ammonia Piranha, the surface layer is removed very quickly and we cannot observe changes in the etching mechanism.

From the thermodynamic principle, UFG materials have lower dissolution potential and therefore a higher tendency of dissolution in aggressive media than coarse-grained counterparts because the former has high density of grain boundaries and higher internal energy. Really, our results suggest higher activity of UFG titanium compared to CG analogs. The values of topography parameters (*R*_a_, RMS, *R*_max_, and *S*_surf_), that characterize development and roughness of the surface, are higher for UFG titanium. This is valid for both types of etching media.

At present, there is not enough information about the chemical etching of UFG titanium. Most known publications are dedicated to etching of only CG titanium [13,14,15,16]. Moreover, most of them are exceptionally experimental. There are no discussions about possible reasons and mechanisms of the formation of one or another relief and surface composition. Therefore, there is a great interest in the works that studied the corrosion (electrochemical etching) of titanium. Some of them are dedicated to the comparison of corrosion resistance of UFG and CG titanium [17]. The data of various authors are contradictory. So, Garbacz et al. [27] suggest that CG titanium is more corrosion resistant compared to UFG analogs. In turn, other authors [18,19,28] report that UFG titanium has a lower tendency to corrode than CG one. It is worth noting that UFG titanium studied in the above-mentioned works was produced using different technologies: hydrostatic extrusion [27], equal channel angular pressing [19,28], and high ratio differential rolling [18]. The difference in the techniques of UFG titanium production led to the difference in the grain size and microstructures. These factors can significantly influence on the chemical and electrochemical stability of the material. Besides, there was a difference in the testing procedures. Garbacz et al. [27] and Hoseini et al. [28] used NaCl-based electrolyte whereas Kim et al. [18] and Balyanov et al. [19] were used HCl and H_2_SO_4_ solutions.

**Figure 11:**
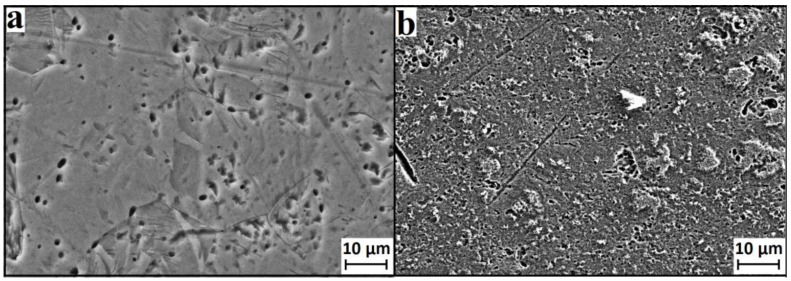
Characteristic SEM images of CG titanium (**a**) and UFG titanium (**b**) etched in H_2_SO_4_/H_2_O_2_ solution during 24 h (magnification—3000×).

It should be emphasized, that UFG materials not only have smaller grain size, but also specific structure on the grain boundaries. Therefore, the main factors determining the corrosion rate of UFG and CG materials are not only grain size, but also volume of grain boundaries, value of internal stress, and the value of electrode potential [17]. Crystal orientation also has a significant impact on etch rate and topography of surface. Matykina et al. [20] and Hoseini et al. [28] demonstrated that crystallographic texture can significantly influence the corrosion rate of UFG and CG Ti. Moreover, it is shown in [28] that texture can play greater role than grain size. In the current work, we did not study crystallographic orientation of the samples in detail, however, we can suppose that while etching in H_2_SO_4_/H_2_O_2_ for a short time the factor of crystallographic texture should not play significant role. This is because of slow etching and low rate of surface layer removal (small mass loss). At longer treatment times we can see (Figure 11) that material grains start to appear and the crystallographic texture factor can be more important. While NH_4_OH/H_2_O_2_ etching, surface layer is removed very quickly and the crystallographic texture factor can play the crucial role. Due to significant influence of the electrode factor, electrochemical etching differs from purely chemical etching. Nevertheless, the influence of internal stress, grain size, and number of grain boundaries is very important for chemical etching.

The difference in etch mechanisms and growth rates of titanium in acidic/basic medium is mainly determined by a difference in chemistry. Chemistry of Ti etching in Piranha solutions is rather complicated. There may be a variety of chemical reactions where Ti and Piranha solution components are involved (oxidation leading to oxides TiO_2_, Ti_2_O_3_, TiO, complex formation in the presence of hydrogen peroxide—Ti(OH)_2_O_2_, hydrogen peroxide/ammonia—Ti(OH)_2_O_2_, formation of low stability sulfates Ti(SO_4_)_2_, TiOSO_4_ etc.) [29,30]. In order to discuss the mechanisms from the point of view of chemical processes during etching in detail, one needs to perform a series of additional time-consuming and thorough experiments using chemical analysis techniques.

It is worth noting that etching can lead not only to material removal, but also to passivation due to surface oxidation. According to XPS and ellipsometry, passivation really takes place while using acidic Piranha solution. Therefore, etching, depending on the conditions, should lead either to the removal of grain boundaries and appearance of the material grains (Figure 11), or to their passivation. Such etching mechanisms can really explain a number of produced nanostructures (“sponge”, “coral”, “mesh”, and “ridges”). Quantitative difference of morphology of these structures for UFG and CG titanium is caused by the difference in the structure and density of grain boundaries.

Another feature of treated samples at prolonged etching is the presence of pits of the size of several microns. Micrographs suggest that their appearance is not related to material grain size and grain boundaries. Probably, pit formation can be explained by the presence of structure and composition defects. It is known that SPD treatment that was used to produce UFG titanium samples leads to redistribution of chemical inhomogeneity into a finer scale. Therefore, higher homogeneity of the structures formed while etching UFG titanium (especially, micron-sized pits) is caused by a more homogenous distribution of impurities.

### 3.2. Prospects for the Use of Materials in Medicine

It was established that basic and acidic Piranha solutions lead, depending on the etching conditions, to the structures of various relief and morphology. The structure size can lie either in micro or in nanoscale. Based on the numerous in vitro and in vivo studies, the researchers agree that the developed relief both on a micro- and nanoscale is necessary for the most successful osteointegration of the implant [24,31,32]. Micron-sized relief, especially the presence of micropores, significantly enhances the adhesion of bone tissue cells [24,33]. The presence of specific nanorelief is very important for enhancement of the circulation and for acceleration of biomolecules (proteins, nutrients) adsorption [24,34], and it can potentially lead to antibacterial effects [35,36]. Besides, from a biomechanical viewpoint, the expanded surface area of the implant surface, which is in contact with the surrounding bone tissue, increases the friction coefficient and the kinetic friction during implant insertion. Ultimately, the increased kinetic friction naturally provides higher implant primary stability [24].

Summarizing aforementioned, the most prominent pathway is surface etching in NH_4_OH/H_2_O_2_ for 2 h, because in this case the samples have the maximal specific surface area, and also developed relief both in nano-ridges and microscale pits. The surfaces of samples etched in H_2_SO_4_/H_2_O_2_ for long periods (from 6 to 24 h) are also characterized by micro- and nanorelief. However, their specific surface area is not so large. Moreover, we can propose that due to higher activity while etching and, correspondingly, due to higher specific surface area, UFG titanium has an advantage over CG analogs.

Surface composition is also very important for a successful medical implant. The most valuable parameter is the presence of a rather thick layer of titanium oxide that prevents biocorrosion and metallic Ti diffusion. Another important parameter is the presence of a large amount of hydroxyl groups on the surface that provide hydrophilic properties of the material [1,2]. This causes faster adsorption of proteins and influences cell activity [37]. From this viewpoint, the advantages of ammonia etchant are not so evident. Despite the fact that etching in NH_4_OH/H_2_O_2_ leads to increasing concentration of surface functional groups and the total amount of these groups is much higher than on the surface of untreated titanium or acidic Piranha etched titanium, oxide layer thickness remains almost unchanged. Besides, experimental data suggest that surface species composition is not sufficiently proved; and the surface can contain not only hydroxyl groups but also some groups of another nature. Nevertheless, the disadvantages of the surface composition produced by chemical etching can be compensated by additional surface modification [12,13,23,38].

## 4. Materials and Methods

### 4.1. Sample Preparation

Nanotitanium samples were prepared in Limited Liability Company “Nanomet”, Ufa, Russia, from titanium Grade 4. Titanium rods of 1 m length were subjected to Equal-Channel Angular Pressing by ECAP-Conform processing. Detailed description of ECAP-Conform processing technique can be found in [6]. Temperature of processing was 400 °C. Number of passes was five. The value of total accumulated true strain was equal to 3.5. After ECAP-Conform processing, the billets were subjected to drawing at 200 °C resulting in the production of rods with a diameter of 6 mm. The average grain size of nanotitanium was ~50–100 nm according to Rietveld XRD processing.

Rods were treated by machining before etching. Firstly, nanotitanium rods were cut into discs having thickness of 2–3 mm by the Buehler IsoMet 1000 machine (Buehler, Lake Bluff, IL, USA). Then, these discs were ground and polished by a semiautomatic Buehler MiniMet 1000 machine (Buehler, Lake Bluff, IL, USA) to mirror-like surface (roughness less than 10 nm) using 600, 800, and 1200 grit sandpapers and silicon dioxide nanoparticles suspension (20 nm). Prior to etching, the samples were cleaned repeatedly with acetone and deionized water in an ultrasonic bath for 15 min and subsequently dried in a desiccator.

Finally, the samples were dropped into a Pyrex glass container with basic (NH_4_OH/H_2_O_2_) or acidic (H_2_SO_4_/H_2_O_2_) Piranha solution at 20 °C. Temperature was maintained by thermostat Elmi TW-2.03. Piranha solutions were prepared by 50% *v*/*v* ammonium hydroxide (NH_4_OH; Vecton, Saint-Petersburg, Russia), 36 N sulfuric acid (H_2_SO_4_; Vecton, Saint-Petersburg, Russia), and 30% aqueous hydrogen peroxide (H_2_O_2_; Vecton, Saint-Petersburg, Russia). The ratio of reactants was 7/3; exposure times were 5, 15 min, and 1, 2, 6, and 24 h for both types of etchants. Immediately after etching, the samples were taken out of the etchant and thoroughly washed in distilled water using an ultrasonic bath.

### 4.2. Samples Characterization

The topography of the samples surfaces was studied using a Solver P47 Pro (NT-MDT, Moscow, Russia) probe microscope in the tapping mode via atomic force microscopy (AFM). The measurements were conducted in ambient air with scan areas of 50 × 50, 30 × 30, 10 × 10, and 1 × 1 μm^2^. A total of five or six random positions on the sample surface were measured. Four parameters including the average mean value of surface roughness (*R*_a_), root mean square roughness (RMS), surface area difference (the percentage increase of 3D surface area over 2D surface area.), and vertical range were calculated by the associated Gwyddion 2.37 software. AFM surface parameters were calculated for all scans and the average values were obtained.

Untreated and treated samples were imagined with scanning electron microscope Zeiss Merlin operated at 10–15 kV at the “Nanotechnology” Interdisciplinary Resource Center SPbSU. Microscope spatial resolution was around 1 nm and magnification up to 600,000×. In-lens, SE and SE2 regimes were used. A total of two or three random positions on the sample surface were scanned.

Chemical composition of the samples before and after etching was study by energy dispersive X-ray fluorescence spectrometer EDX Series 800 HS (Shimadzu, Kyoto, Japan). Chemical composition of the samples surface was study by X-ray photoelectron spectroscopy (XPS). X-ray photoelectron spectra were registered with a “Thermo Fisher Scientific Escalab 250Xi” spectrometer (Thermo Fisher, Waltham, MA, USA) at the Resource Center of “Physical Methods of Surface Investigation” SPbSU. The samples were excited by Al Kα (1486.7 eV) X-rays in a base pressure of 7 × 10^−8^ Pa. High resolution spectra were automatically charge compensated by setting the binding energy of C 1s carbon line to 284.8 eV [39].

The thicknesses of oxide layers on the surface of the samples were estimated by spectral ellipsometry (350–1000 nm) method using Ellips-1891 SAG instrument (CNT, Novosibirsk, Russia). Accuracy of the film thickness determination was 0.3 nm in a thickness range of 1–100 nm

## 5. Conclusions

In this study, we investigated in detail the features of the etching of ultrafine grain (UFG) and coarse grain (CG) titanium in Piranha solutions (NH_4_OH/H_2_O_2_ and H_2_SO_4_/H_2_O_2_). Using AFM and SEM methods, it was found that the variation of the etching medium and time leads to various micro-, nano-, and hierarchical micro/nano-structures on the UFG or CG titanium surface. AFM results suggest that ammonia Piranha solution (in contrast to the acidic one) provides more significant roughness and value of specific surface area even at small etching times. H_2_SO_4_/H_2_O_2_ Piranha solution gives a meaningful increase of relief parameter only after 2 h etching. Based on XPS and ellipsometry data, we suggest that the difference is caused by a less distinct oxidation (passivation) process in the basic Piranha solution compared to the acidic one. UFG titanium surface composition is different after etching in NH_4_OH/H_2_O_2_ and H_2_SO_4_/H_2_O_2_ solutions.

SEM and AFM data showed that UFG titanium is more actively etched compared to CG titanium independently of the etching medium. The difference is both qualitative (variation of surface morphology and homogeneity) and quantitative (roughness and specific surface area). The causes of these differences can be either variations of the materials structures (grain size, amount, and the structures of grain boundaries) or impurities distribution.

We proposed that UFG titanium samples etched in NH_4_OH/H_2_O_2_ for 2 h and in H_2_SO_4_/H_2_O_2_ for 24 h can be very prominent materials for dental and orthopedic implants due to well-developed surface and presence of hierarchal micro/nano structures on the surface.

## Supplementary Materials

The following are available online at http://www.mdpi.com/2079-4991/7/1/15/s1.
