# Supplementary material for: Modification of the Surface Topography and Composition of Ultrafine and Coarse Grained Titanium by Chemical Etching
Source: Nanomaterials (Basel). 2017 Jan 13;7(1):15. doi: 10.3390/nano7010015 (PMC5295205; doi:10.3390/nano7010015)
Supplement: Supplementary file 1 [file nanomaterials-07-00015-s001.pdf]

Supplementary Materials: Modification of the Surface Topography and Composition of Ultrafine and Coarse Grained Titanium by Chemical Etching

Denis V. Nazarov, Elena G. Zemtsova, Alexandr Yu. Solokhin, Ruslan Z. Valiev and Vladimir M. Smirnov

Table S1. Topography parameters of the ultrafine grained (UFG) titanium surface etched in H_2SO_4/H_2O_2 solutions.

Scan Area, µm	Etching Time	R_{max}, nm	R_a, nm	RMS, nm	S_{surf}
1 × 1	0	17.1 ± 3.1	1.35 ± 0.17	1.91 ± 0.19	1.023 ± 0.004
	5 min	18.0 ± 2.9	1.46 ± 0.18	2.01 ± 0.22	1.031 ± 0.005
	15 min	17.8 ± 3.4	1.10 ± 0.15	1.51 ± 0.16	1.040 ± 0.004
	1 h	17.2 ± 1.9	1.51 ± 0.10	1.92 ± 0.12	1.015 ± 0.002
	2 h	16.5 ± 3.5	1.17 ± 0.23	1.52 ± 0.24	1.021 ± 0.004
	6 h	71.4 ± 11.9	5.14 ± 1.00	6.93 ± 1.10	1.040 ± 0.007
	24 h	49.3 ± 7.8	4.00 ± 0.80	5.41 ± 0.85	1.060 ± 0.006
10 × 10	0	126 ± 11	3.62 ± 0.67	6.54 ± 0.79	1.013 ± 0.003
	5 min	109 ± 13	3.77 ± 0.58	6.43 ± 0.92	1.012 ± 0.003
	15 min	125 ± 13	3.55 ± 0.55	6.18 ± 0.76	1.010 ± 0.002
	1 h	95.7 ± 9.9	5.04 ± 0.50	6.85 ± 0.59	1.012 ± 0.004
	2 h	142 ± 14	3.50 ± 0.73	4.93 ± 1.04	1.014 ± 0.003
	6 h	474 ± 42	21.5 ± 4.2	31.9 ± 5.1	1.071 ± 0.007
	24 h	601 ± 48	37.8 ± 3.8	53.1 ± 6.5	1.065 ± 0.006

Table S2. Topography parameters of the ultrafine grained (UFG) titanium surface etched in NH_4OH/H_2O_2 solutions.

Scan Area, µm	Etching Time	R_{max}, nm	R_a, nm	RMS, nm	S_{surf}
1 × 1	0	17.1 ± 3.1	1.35 ± 0.17	1.91 ± 0.19	1.023 ± 0.004
	5 min	57.7 ± 2.9	7.31 ± 0.16	9.29 ± 0.24	1.044 ± 0.014
	15 min	78.1 ± 3.9	7.58 ± 0.25	9.68 ± 0.23	1.402 ± 0.011
	1 h	97.9 ± 7.5	9.96 ± 0.56	12.7 ± 0.6	1.561 ± 0.015
	2 h	97.7 ± 5.2	11.3 ± 0.5	14.3 ± 0.5	1.419 ± 0.019
	6 h	96.4 ± 4.6	10.5 ± 0.4	13.5 ± 0.8	1.206 ± 0.017
	24 h	80.4 ± 5.0	8.64 ± 0.49	11.0 ± 0.6	1.328 ± 0.012
10 × 10	0	126 ± 11	3.62 ± 0.67	6.54 ± 0.79	1.013 ± 0.003
	5 min	199 ± 15	13.5 ± 1.1	17.6 ± 1.9	1.099 ± 0.012
	15 min	476 ± 40	40.6 ± 4.3	52.6 ± 5.2	1.151 ± 0.013
	1 h	486 ± 29	33.7 ± 3.6	45.5 ± 5.1	1.154 ± 0.023
	2 h	912 ± 37	63.8 ± 4.3	80.2 ± 5.2	1.350 ± 0.021
	6 h	1037 ± 69	95.0 ± 9.0	127 ± 13	1.280 ± 0.034

24 h	888 ± 71	75.3 ± 6.8	103 ± 7	1.117 ± 0.021

Table S3. Topography parameters of the coarse grained (CG) titanium surface etched in H_2SO_4/H_2O_2 solutions.

Scan Area, μm	Etching Time	R_{max}, nm	R_a, nm	RMS, nm	S_{surf}
1 × 1	0	19.6 ± 1.4	1.84 ± 0.17	2.33 ± 0.21	1.029 ± 0.002
	5 min	22.3 ± 1.8	1.98 ± 0.19	2.78 ± 0.24	1.021 ± 0.002
	15 min	25.6 ± 2.9	2.23 ± 0.28	2.90 ± 0.31	1.027 ± 0.003
	1 h	21.1 ± 1.0	1.58 ± 0.09	2.12 ± 0.12	1.013 ± 0.002
	2 h	22.7 ± 1.3	2.30 ± 0.10	2.90 ± 0.16	1.017 ± 0.002
	6 h	36.0 ± 2.9	3.39 ± 0.35	4.33 ± 0.51	1.057 ± 0.007
	24 h	46.4 ± 7.7	3.13 ± 0.47	4.24 ± 0.62	1.030 ± 0.004
10 × 10	0	96.0 ± 4.2	4.84 ± 0.35	6.33 ± 0.46	1.0089 ± 0.0006
	5 min	86.0 ± 3.7	4.55 ± 0.33	6.45 ± 0.55	1.0101 ± 0.0009
	15 min	80.1 ± 6.3	4.68 ± 0.26	6.13 ± 0.31	1.0082 ± 0.0006
	1 h	149 ± 23	6.45 ± 0.89	10.0 ± 1.6	1.0107 ± 0.0009
	2 h	82.8 ± 39.0	5.00 ± 0.32	7.05 ± 0.41	1.0054 ± 0.0005
	6 h	343 ± 45	17.1 ± 2.2	24.4 ± 3.1	1.058 ± 0,005
	24 h	329 ± 44	20.3 ± 2.4	28.4 ± 3.5	1.051 ± 0,005

Table S4. Topography parameters of the coarse grained (CG) titanium surface etched in NH_4OH/H_2O_2 solutions.

Scan Area, μm	Etching Time	R_{max}, nm	R_a, nm	RMS, nm	S_{surf}
1 × 1	0	19.6 ± 1.4	1.84 ± 0.17	2.33 ± 0.21	1.029 ± 0.002
	5 min	102 ± 7	7.84 ± 1.30	9.37 ± 0.81	1.030 ± 0.003
	15 min	105 ± 9	9.22 ± 0.78	11.9 ± 0.9	1.323 ± 0.027
	1 h	94.8 ± 4.7	10.0 ± 0.5	12.7 ± 0.7	1.158 ± 0.016
	2 h	77.3 ± 8.2	8.14 ± 0.91	10.5 ± 1.3	1.088 ± 0.007
	6 h	170 ± 20	19.1 ± 2.7	24.6 ± 3.2	1.276 ± 0.023
	24 h	70.5 ± 9.7	7.71 ± 1.1	10.0 ± 1.8	1.069 ± 0.005
10 × 10	0	96.0 ± 4.2	4.84 ± 0.35	6.33 ± 0.46	1.0089 ± 0.0006
	5 min	289 ± 11	19.4 ± 1.0	25.8 ± 1.2	1.0686 ± 0,0043
	15 min	303 ± 26	17.2 ± 1.3	23.9 ± 1.8	1.079 ± 0,005
	1 h	368 ± 21	27.6 ± 1.8	36.3 ± 1.9	1.098 ± 0,012
	2 h	830 ± 76	67.0 ± 7.0	95.1 ± 9.9	1.130 ± 0,016
	6 h	830 ± 69	68.7 ± 5.2	87.7 ± 6.1	1.299 ± 0,031
	24 h	690 ± 38	56.1 ± 2.8	77.8 ± 4.1	1.087 ± 0,004
